# HIV Stigma and Moral Judgement: Qualitative Exploration of the Experiences of HIV Stigma and Discrimination among Married Men Living with HIV in Yogyakarta

**DOI:** 10.3390/ijerph17020636

**Published:** 2020-01-19

**Authors:** Dionius B. Mahamboro, Nelsensius K. Fauk, Paul R. Ward, Maria S. Merry, Theodorus A. Siri, Lillian Mwanri

**Affiliations:** 1 Faculty of Theology, Sanata Dharma University, Yogyakarta 55283, Indonesia; dionius.bismoko@usd.ac.id; 2 College of Medicine and Public Health, Flinders University, GPO Box 2100, Adelaide 5001, South Australia, Australia; fauk0001@flinders.edu.au (N.K.F.); lillian.mwanri@flinders.edu.au (L.M.); 3 Institute of Resource Governance and Social Change, Kupang, Nusa Tenggara Timur 85221, Indonesia; 4 Medicine Faculty, Duta Wacana Christian University, Yogyakarta 55224, Indonesia; silvia.tropmed@gmail.com; 5 Saint Peter Pastoral Institute of the Diocese of Atambua, Nusa Tenggara Timur 58163, Indonesia; theoasasiri@yahoo.com

**Keywords:** HIV stigma and discrimination, moral judgement, HIV-positive men married to women, Yogyakarta, Indonesia

## Abstract

It is well acknowledged that human immunodeficiency virus stigma (HIV stigma) challenges people living with HIV globally. There is a scarcity of information about determinants of HIV stigma and discrimination among married men in the Indonesian context. This study aimed to explore factors that contribute to stigma and discrimination against HIV-positive men married to women in Yogyakarta, Indonesia. Face-to-face in-depth interviews were conducted to collect data from participants using a snowball sampling technique. A framework analysis was used to guide the analysis of the data. HIV stigma framework was also applied in the conceptualisation and the discussion of the findings. The findings indicate that participants experienced external stigma within healthcare facilities, communities and families. This external stigma was expressed in various discriminatory attitudes and behaviours by healthcare professionals and community and family members. Similarly, participants experienced anticipated stigma as a result of HIV stigma and discrimination experienced by other people living with HIV. Individual moral judgement associating HIV status with amoral behaviours and participants’ negative self-judgement were determinants of perceived stigma. The current findings indicate the need for training programs about HIV stigma issues for healthcare professionals. There is also a need to disseminate HIV information and to improve HIV stigma knowledge among families and communities.

## 1. Introduction

Globally, human immunodeficiency virus stigma (HIV stigma) and discrimination affect physical and mental health of people living with HIV (PLHIV) [1,2]. HIV stigma and discrimination against PLHIV have been demonstrated in various forms, such as negative labelling, discriminatory behaviours and negative treatment by healthcare professionals, families and communities [3,4]. It is also known that HIV stigma and discrimination have negative impacts on PLHIV, such as self-isolation, social exclusion, loss of jobs, economic difficulty and poor access to healthcare services [2,5,6].

Studies in different settings have identified the determinants of HIV stigma and discrimination against PLHIV, including the lack of basic knowledge of the route of HIV transmission and there is also a belief that being infected with HIV is an individual responsibility [2,3]. In addition, poor understanding of effective HIV current management has led to the perception that HIV is still a life-threatening infection. Studies by Liu and Tsui [7] and Liu and colleagues [8] suggested that people who have poor HIV/AIDS knowledge are more likely to hold negative attitudes and behaviours toward PLHIV. Further studies [2,9,10] have also reported community fears and discomfort while interacting with PLHIV as being determinants of HIV stigma and discrimination. Fear, shame, and blame in many cultures build irrational beliefs (e.g., physical contact with PLHIV can transmit the virus) about HIV transmission and cause stigma and discrimination against PLHIV [6,11]. The perceptions that AIDS is a disease of men who have sex with men (homosexuality) and that HIV infection is associated with amoral sexual behaviours (e.g., having sex with multiple sex partners or sex work) increase and maintain stigma and discrimination against PLHIV [12,13,14,15].

Several studies in Indonesia have focused on HIV-related stigma and discriminatory attitudes by healthcare professionals and identified factors including poor knowledge on HIV transmission and prevention modalities [16,17,18,19], religious perceptions that HIV is a punishment from God and a belief that HIV positive status is a rejection of homosexuality [20]. Studies in other settings have focused on HIV-positive women and men who have sex with men and identified gender, sexual orientation [21,22,23,24,25,26,27] and other factors as supportive of HIV stigma and discrimination [28,29,30,31].

In Indonesia, men married to women make up the majority of adult men in communities and make up a significant proportion of PLHIV [32]. However, no studies have attempted to specifically and qualitatively explore the views of ‘married men’ living with HIV on factors associated with HIV stigma and discrimination in Indonesia. This paper fills the gap in knowledge on the evidence of determinants of HIV stigma and discrimination among ‘married men’ in the Indonesian context. The understanding of these factors is important to inform the development of policies and programs that address the problem among men married to women and other PLHIV in Indonesia. This qualitative exploratory study aimed to understand determinants of HIV stigma and discrimination against HIV-positive men married to women in Yogyakarta, Indonesia.

## 2. Methods

### 2.1. Study Setting

The city of Yogyakarta, the current study’s focal setting, is a municipality in the Special Region of Yogyakarta province. The city is divided into 14 sub-districts and 45 villages and has a total population of 422,732 people (13,007.13 people per km^2^) [33]. The majority of people in Yogyakarta are cultural groups, including Javanese (the main group comprising about 87% of total population), Sundanese, Malay, Chinese, and Batak [32]. It is the capital city of the Special Region of Yogyakarta province, which is one of the 34 provinces in Indonesia. This province is situated in the south of Java Island, covers an area of 3158.80 km^2^, and has the total population of 3,452,390 people [33]. The number of HIV cases in Yogyakarta has been reported to be increasing annually, from 4060 in 2016 to 4783 in 2017, 5616 in 2018 and 5891 in June 2019, and the prevalence rate of HIV infection up to July 2019 was 0.0017%, which was higher than that of the national rate of 0.0013% [32]. The municipality of Yogyakarta, where the current study was conducted, provides HIV-related health services in four hospitals and 10 community health centres. The services provided include dissemination of health information on HIV/AIDS and the related services, HIV counselling and testing, CD4 tests, viral load tests, and ART provision [34,35,36]. In addition, several non-governmental organisations in collaboration with the health sector in the study setting provide HIV-related support, such as regular HIV information sessions and mobile voluntary counselling and testing for target groups and general communities, peer support groups for PLHIV, monthly meetings with PLHIV and sustained support to ensure access and adherence to treatment among PLHIV [35]. Yogyakarta is one of the few cities in Indonesia where HIV-related information and health services have been reported to be relatively well disseminated and provided [37,38]. Understanding HIV stigma and discrimination in the context of the current study setting is necessary to inform practices and policies for improvements in HIV management. Evidence from the current setting can also be used to inform further investigation of this problem in other parts of the country, where these services are not readily available.

### 2.2. Conceptual Framework

The conceptualisation and discussion of the current study findings were guided by the HIV stigma framework by Earnshaw and Chaudoir [39]. The framework suggests that HIV stigma is a devalued attribute which has negative impacts on both uninfected and infected people within a given society through its concomitant stigma mechanisms. Stigma mechanisms reflect how people respond to the knowledge or the fact that they either have (HIV infected) or do not have (HIV uninfected) the devalued attribute. For uninfected people, stigma mechanisms reflect the psychological responses to the knowledge of the existence of HIV-infected people who may transmit the virus to them or threaten their life and health and who possess moral blemishes [40]. Such responses are often reflected in efforts to avoid people who are infected with HIV [41]. There are three predominant ways through which stigma mechanisms are manifested, which show how uninfected people react towards infected people: prejudice, stereotyping, and discrimination towards PLHIV. Prejudice represents negative emotions of uninfected people towards and how they feel (such as disgusted, angry, and afraid) about PLHIV [42]. Stereotypes (e.g., that PLHIV are sexually promiscuous or of low moral standing) refer to the beliefs held by a certain group of people or community about PLHIV that are often applied to specific individuals living with HIV [43]. Discrimination reflects the behaviours of uninfected people towards PLHIV as the expression of prejudice [42]. For PLHIV, stigma mechanisms reflect the psychological responses to the knowledge or fact that they may have violated social mores and religious norms and values, and thus may be treated negatively by other people. This framework suggests that stigma mechanisms experienced by PLHIV (enacted stigma, anticipated stigma and internalised or perceived stigma) can cause negative impacts on their behaviours, psychological state, social life and health outcomes. Enacted stigma is about the belief that PLHIV have that they have actually experienced prejudice and discrimination from other people [44]. Anticipated stigma reflects the degree to which PLHIV expect that they will experience prejudice and discrimination from other people [45]. Perceived stigma reflects the individual’s attitudes and behaviours of others and internalised stigma reflects the degree to which PLHIV endorse the negative beliefs and feelings associated with HIV about themselves [46].

### 2.3. Study Design

A qualitative inquiry employing one-on-one face-to-face in-depth interviews was conducted from May to August 2018 to explore married men’s experience of HIV stigma and discrimination in Yogyakarta. The use of qualitative design has been found appropriate to explore participants’ understanding, interpretations, values and meanings regarding HIV stigma and discrimination facing them in their daily lives [47,48,49], and is effective when exploring participants’ perspectives and deep insight of their real life experiences [48,50].

### 2.4. Recruitment and Data Collection

The study participants (n = 20) were HIV-positive men married to women in the city of Yogyakarta, Indonesia. They were recruited using the snowball sampling technique [48]. The study information sheets with the contact details of two male field researchers (NKF and DBM) were initially posted on the information board of a non-governmental organisation providing support and services for PLHIV. Participants who contacted the researchers and agreed to take part in the study were recruited. At the end of each interview, the participants were asked to disseminate the study information sheets to other potential participants, including their friends and colleagues who might be willing to take part in the study. This recruitment process was continued until the researchers felt that the data were rich enough and data saturation had been reached [51]. A total of 20 participants were recruited based on the inclusion criteria that one had to be 18 years old or above, married, and HIV-positive. No one other than the researcher and participant was present in the interview room. None of the potential participants who agreed to be interviewed withdrew their participation. The interviews were conducted in Bahasa.

Face-to-face in-depth interviews were conducted at a time and place agreed upon by the researcher and participant. Each potential participant who called and stated his willingness to be interviewed was offered a possible date and place for interview. The interviews with each participant were conducted in a private research room at Duta Wacana Christian University. The interview focused on exploring the HIV-related stigma and discrimination experience of the participants within families, communities, and healthcare settings, and their own perceptions about their HIV status. The main areas of questioning in the interview included: the attitudes and treatments of healthcare providers or doctors and nurses they had received in any healthcare facilities; the attitudes and reactions of other community members who knew about their HIV status; participants’ daily experience of the attitude and treatment of other family members after HIV diagnosis; participants’ perceptions on how other people would treat or react to their HIV status, if known; and the feelings and perceptions the participants had about themselves in relation to their HIV status. The duration of each interview ranged from 45 to 60 min. At the end of the interview, each participant was offered the opportunity to read and correct the transcript once it has been transcribed; however, no participants chose to do so.

### 2.5. Ethical Consideration

Ethics approval for this study was obtained from the Duta Wacana Christian University health research ethics committee (No. 640/C.16/FK/2018). Prior to the interviews, each participant was informed about the purpose of the study and that the study had obtained ethical approval. They were also advised about the voluntary nature of their participation and that they had the right to withdraw their participation without any consequences if they felt uncomfortable with the questions being asked. They were also advised that the interview would take approximately 45 to 60 min and would be recorded using a digital tape recorder. They were assured that the data or information that they provided during the interview was confidential and anonymous, as each participant was assigned with a specific study identification letter and number. This was to prevent the possibility of linking back the data or information to any individual in the future. Each participant received reimbursement of IDR 100,000 (±USD 7) for the transport and time spent. Before commencing the interviews, each participant signed the informed consent form and returned it to the researcher.

### 2.6. Data Analysis

The recorded interviews were transcribed into coding sheets and translated into English by the authors (NKF and DBM) who are fluent in Bahasa and English. To maintain the quality and validity of the data, data cross checking was performed by the authors during the transcription and translation processes. The translations were also checked for accuracy by other authors. The transcripts were imported into the qualitative data analysis software package ATLAS.ti version 8.4.3 (Scientific Software Development GmbH, Berlin, Germany). The data analysis was guided by Ritchie and Spencer’s framework analysis [52]. This framework suggests six steps in qualitative data analysis. First, familiarisation with the data through the repeated reading of the transcripts, taking notes, and giving comments and labels to the transcripts; second, identification of a thematic framework where recurrent issues, concepts and themes were written down; third, indexing the entire data by creating a list of codes (open coding) to search for similar and redundant codes to reduce long list of codes into a manageable number. This was followed by closed coding where similar codes or codes that referred to the same theme were grouped together; fourth, charting the data by arranging appropriate thematic references in a summary chart which enabled the researchers to compare the data across the interviews and within each interview; fifth, mapping and interpretation of the data through which the ideas that made each theme were examined to see the relationship and association between them [52].

## 3. Results

### 3.1. Characteristics of the Participants

The participants’ age ranged from 27 to 51 years, with the mean age of 40.9 years. All the participants resided in the city of Yogyakarta, 19 were Javanese and one was Chinese. They had different levels of educational background (see Table 1). More than a half (n = 12) acquired HIV infection through injecting drug use, while the others got infected through unprotected sexual intercourse with multiple female sex partners (n = 3) and multiple female and male sex partners (n = 5). Five people had been HIV positive for 2–5 years, 11 people for 6–10 years and four people for more than 10 years.

### 3.2. External or Enacted Stigma

#### 3.2.1. Stigma and Discrimination from Healthcare Providers

Participants described having experienced HIV-related discrimination attitudes from healthcare professionals within healthcare settings. For example, they commented that they were left untreated for many days in healthcare facilities (hospitals) while doctors and nurses seemed unwilling to help them during their stay in the hospitals:

“…. The first time I was admitted to hospital X [pseudo name], I was left untreated at all, I hardly breathed at the time, the doctor knew that, but he did not do anything to help me. I did not receive any medical treatment for 10 days in the hospital” (R10, 48 years old).

“The problem with B20 patients like me is that the nurses, once our status is known to them [doctors and nurses], they seemed reluctant or unwilling to help us get better. They seemed lazy to treat me when I was in the hospital” (R13, 44 years old).

“…. I once experienced discriminatory treatment in the hospital, the doctor did not want to be close to me. He seemed very afraid to replace the infusion” (R18, 38 years old).

Discriminatory treatment from healthcare professionals created negative impressions and perceptions among PLHIV towards healthcare professionals. The participants had the perception and impression that healthcare professionals seemed to feel disgusted by their health conditions and treated them in unfriendly ways:

“…. The nurse who helped me at that time was so unfriendly. I was not comfortable at all. I felt that the nurse felt disgusted by my condition and did not want to touch me, the infusion hose was also thrown in front of me, so rude. There were a few nurses who I knew that they felt disgusted with HIV patients (R9, 27 years old).

“…. I once underwent blood check at a private laboratory clinic, the nurse wore disposable gloves, but after that she went back and forth, back and forth a few times to wash her hands. Oh my God…. should it be like that? It felt like I was disgusting” (R10, 48 years old).

Discriminatory behaviours led to perceptions among the participants that some healthcare professionals were not well informed about HIV and how to treat patients with HIV:

“…. The doctor did not want to treat me and was scared to change the infusion bottle. I was mad at the doctor, how come a medical doctor does not know information about HIV? They should know how to treat patients with HIV like me and do not leave patients with HIV untreated” (R8, 38 years old).

“I think healthcare professionals who are not specifically trained about HIV lack information and knowledge about HIV. The treatments from those who work in HIV clinic and the ones who do not are different” (R1, 29 years old).

There was also a mistrust by patients that healthcare professionals were unable to keep confidentiality about patients’ HIV status:

“There are healthcare professionals who spread HIV status of patients: ‘This patient is infected with HIV, be careful …. I think they are the ones who are not well informed about HIV” (R4, 42 years old).

Discriminatory behaviours against the participants within healthcare settings were reported to influence their access to healthcare services. Several participants commented that they were reluctant to access healthcare services in healthcare facilities where they had previously experienced discriminatory treatments from nurses or doctors. As a consequence, some decided to access healthcare services in other healthcare facilities:

“…. I felt uncomfortable with the treatment of the doctor that handled me, so I was reluctant to go to the same hospital, I may meet her again” (R7, 34 years old).

“I was ashamed and asked myself why did they treat me so rude? …. After that I do not go the place [healthcare facility] anymore. …. I go to another community health center” (R16, 30 years old).

“…. I do not want to be served by the same person [nurse] who has treated me unfriendly, so I do not go there anymore” (R20, 39 years old).

#### 3.2.2. Stigma and Discrimination from Community Members

HIV stigma and discrimination against the participants were reported to occur within communities where they lived and interacted. Refusing to sit close to PLHIV or moving away from the chairs next to PLHIV, keeping distance and fear of having direct physical contact with PLHIV were the instances of discriminatory behaviours by other community members:

“…. There are community members who keep their distance from me, this is very obvious. They moved away from me to other chairs and refused to sit next to me” (R4, 42 years old).

“There are some [community members] who said ‘he has this disease [HIV/AIDS], we may get infected’. They did not want to sit next to me at all ….” (R2, 42 years old).

Lack of information and knowledge about the means of HIV transmission was expressed by the participants as a factor which maintains discriminatory behaviour against PLHIV within communities. Participants commented that many community members were not informed about the basic knowledge of HIV, especially modes of HIV transmission. This perception was supported by the fact, as acknowledged by the participants, that dissemination of HIV-related information had not reached many communities in Yogyakarta:

“Discrimination against people living with HIV depends how well-informed community members are about HIV. In some villages it seems like the ratio of people who are not informed and the ones who are informed about HIV is 10:1. HIV-related stigma and discrimination are worse in such villages. They [community members] deem HIV/AIDS as a horrible disease” (P1, 29 years old).

“I am also involved in HIV programs and activities, including HIV information sessions for community members but we have not reached many communities or groups, and many people are not informed about this infection. This is reason why they stigmatise and discriminate people with HIV” (R5, 33 years old).

#### 3.2.3. Stigma and Discrimination from Family Members

HIV-related stigma and discrimination were also reported to occur within families of the participants. Isolation and separation of personal belongings and eating utensils were the examples of discriminatory behaviour or treatment experienced by the participants within their families, performed by other family members, such as their parents and the family of their wives. Several comments of the participants illustrated such experiences:

“When I was physically weak [sick] and admitted to the hospital, I felt like I was left and isolated by my family members: parents and siblings …. I felt isolated because all my personal belongings, eating utensils, and toiletries were separated from those of other family members” (R20, 39 years old).

“… Once I was tested positive with HIV, all the family members of my wife started to keep distance from me. It is so obvious: foods and plates are separated. They also avoid touching my clothes because they think it can be transmitted through sweat” (R3, 31 years old).

However, stigma and discrimination against the participants seemed to be diminished once the family members were informed about the modes of HIV transmission:

“… My parents and siblings were taught about HIV by the doctor who handled me and finally they accept me, I am welcomed in the family…. It is because I was accompanied by my mom once I consulted the doctor about the situation, I told the doctor everything I experienced in the family. The doctor said to my mom: “you do not need to separate eating utensils, they do not transmit the virus ….” I was so happy because after that they [family members] do not separate my eating utensils anymore” (R20, 39 years old).

### 3.3. Anticipated Stigma

Anticipated stigma was prevalent among the study participants. Fear of being discriminated against by other community members, the intention to maintain the reputation of the extended family and to avoid negative impacts on children, and the perception of HIV as a disgrace were the factors maintaining anticipated stigma among the participants. Such anticipated stigma seemed to support the decision of some of the participants to hide their HIV status from community members:

“…. If other people know about my status, would I be accepted? That is what I am scared of. HIV is like a taboo thing for many people, especially people who know nothing about it. So, I keep it secret to secure the reputation of my family and avoid negative impacts on my children. I am afraid if my children are discriminated due to my HIV status” (R11, 43 years old).

“…. I think that getting infected with HIV is my fault, and this is my life. So, I am not sure that other people who I tell them about my HIV status understand and care about me. This infection is disgrace for me, and I think I do not need to let other people know about it” (R16, 48 years old).

Negative experiences of other PLHIV they had seen or heard of, such as being neglected, avoided, rejected and having their personal belongings burned were additional factors linked to anticipated stigma, often leading to non-disclosure of their HIV status. Several participants commented that they did not want to experience the same negative treatments as their friends:

“…. I heard that a friend died of HIV/AIDS and nobody wanted to bathe his body. His family members did not want to …. At the end, other friends who were companions of people with HIV [they are also HIV-positive] took the initiative to bathe the body. I also heard that nobody wanted to carry him to the cemetery because people were scared of being infected. …. So, I am afraid that if I am open about my status, I might experience the same thing” (R9, 27 years old).

“Another friend of mine died from AIDS in Bali, all his personal belongings and sleeping equipment were burned off. The same thing can happen to me as well, so I do not tell everybody about my status” (R1, 29 years old).

“My close friend died, and he was HIV-positive. Everybody in the village avoided and did not want to bathe his body, and even his own family did not want to do that and seemed to reject him. Two other friends of mine and I were the ones who bathed the body ….” (R15, 48 years old).

### 3.4. Perceived Stigma and Participants’ Individual Moral Judgement on their HIV Status

Perceived HIV stigma was also experienced by the participants, which served to maintain their individual moral judgement on their HIV status. Several participants commented that they felt ashamed of their HIV status due to the perceptions that getting infected with HIV was associated with amoral behaviours and engagement in sex with sex workers:

P (Participant): “Before I met other friends who are also HIV-positive I was ashamed of having HIV infection. But now if someone asks and he or she really wants to know then I will explain. But so far, nobody asks”

R (Researcher): How will you explain about it?

P: “I will explain that I get this infection not through sex but injecting drug use”

R: Do you think there is a difference between getting HIV through sex and injecting drug use?

P: “I will be so sad if I get it through sex because people will judge me as a bad person. If I am infected by sex workers, then that means I am a bad guy. But if I am infected through injecting drug use [sharing needle], then it is associated with male delinquency, so I will not think or be ashamed of it” (R10, 48 years old).

“I feel ashamed of having this infection because people must think that I have had sex with sex workers” (R6: 42 years old).

The above perception of HIV/AIDS, which is associated with amoral behaviours and which seems to lead to negative self-judgement as a ‘bad person’, increased guilty feelings and perceived stigma among the study participants. Several of them commented that they felt guilty about having the infection and judged themselves as “dirty people” or sinners:

“…. I feel guilty, why I had done that [having sex with other men], it is forbidden by the religion. So, I do feel guilty because of that, I am a sinner” (R9, 27 years old).

“I feel guilty because of the stigma from the society that HIV/AIDS is the disease of people with dirty [amoral] behaviours. So, I am a dirty person, have a lot of sins” (R6, 42 years old).

“People perceive that HIV/AIDS is the disease of specific groups of people: transgender women, sex workers, and men who have sex with men. People may think that I belong to one of these groups” (R4, 42 years old).

Participants’ moral judgement also seemed to endorse negative stereotypes about themselves or self-stigma. Self or internal stigma can be deduced from the following sentiments expressed by participants:

“I am a sinner” (R9, 27 years old).

“I am a dirty person, have a lot of sins” (R6, 42 years old).

“I am a bad person because I did what I was not supposed to do” (R17, 51 years old).

Two other participants expressed negative stereotypes of themselves or self-stigma which seemed to be influenced by their religious thoughts:

“What I did [sex with multiple sex partners before marriage] was opposite to what I heard from the church, I am a very bad person” (R12, 41 years old).

“At the first time I was diagnosed with HIV, some family members questioned me “how do you get the infection?” And once they knew that I might have got it from any sex workers I had sex with, some said “you should not have done that, it is wrong, it is sin. You have done a wrong thing which is not allowed in our religion. I feel like I am a very guilty person, a sinner. I was long time ago but what they said still stays with me up to now” (R14, 42 years old).

## 4. Discussion

Stigma and discrimination are often experienced by PLHIV due to their HIV-positive status. This study aimed to explore HIV stigma and the discriminatory experiences of HIV-positive men married to women in Yogyakarta, Indonesia. Consistent with previous findings [9,10,53,54,55], this study identified that stigma and discrimination against HIV-positive men married to women came from healthcare profession, family members, their local community and their own self-evaluations.

Healthcare professionals’ discriminatory attitudes and behaviours in the current study manifested in various forms, such as an unwillingness and reluctance to treat/touch patients with HIV, and being unfriendly towards them. As per previous studies [9,10,53,54,55,56], these findings are an indication of health professionals’ poor knowledge of HIV, as they think that they could be infected with HIV through interaction with the patients. The discriminatory treatment experienced by the current study participants was probably conducted by healthcare professionals who were not formally trained in HIV, because in Indonesia, HIV patients who are admitted to general hospitals are taken care of by healthcare professionals from units such as the intensive care unit, who might not be well-equipped with information or knowledge about HIV [57,58]. Consistent with previous studies [59,60,61,62], the participants in this study were reluctant to access healthcare services in healthcare facilities where they had previously experienced discriminatory attitudes or treatment, which is indicative of the need for training programs among health care professionals across facilities. Training of health professional has been identified as effective in addressing HIV stigma, as indicated previously in a study with transgender women in the same study setting, where the availability of positive support by healthcare professionals led to effective access to HIV services by transgender participants [35].

Consistent with other studies [63,64,65], the current findings indicate that HIV stigma and discrimination against the participants occurred within families and communities due to a lack of knowledge about HIV transmission. These findings support the HIV stigma framework and previous findings [39,41,42,66,67], where poor understanding about HIV in general has been stated as one of the main reasons for prejudice, stereotypes and discrimination from family and community members against PLHIV. Similarly, according to the findings elsewhere [68,69,70,71], anticipated stigma was common among the current study participants due to the fear of discrimination, shame to the family and negative impacts on children. In Indonesia, family is deemed as an entity where the behaviours and HIV status of a family member can have serious negative impacts on the extended family [6,72]. This has been underlined in Ho and Mark’s concept [73] asserting that societies that emphasise collectivism, such as in Indonesia, can blame or stigmatise the entire extended family if one among family members is infected with HIV. This seemed to be one of the cultural factors that contributed to anticipated stigma among the study participants, leading to participants concealing their HIV status [74,75,76]. Concealment of HIV status from other community members may also lead to the lack of social support for PLHIV, which has been reported to lead to HIV stigma and discrimination against PLHIV [77,78] and poor access to HIV healthcare services [79], a vicious cycle.

Experiences of perceived stigma were also identified from participants’ own moral judgement on their HIV status. Being infected with HIV was associated with amoral behaviours (e.g., engagement in sex with sex workers), which led to the participants’ self-judgement as ‘dirty people’ or sinners. The HIV stigma framework [39,40] suggests that self-judgement or ‘moral blemishes’ contribute to the internal stigma experienced by PLHIV. This finding also supports the idea that stigma is associated with social and cultural process and a moral issue in which the conditions that are stigmatised (in this case HIV) threaten what is considered valuable to a social group and community [80,81]. Participants’ moral judgement on their behaviours through which they contracted the infection (sex with multiple sexual partners) and self-judgement as “dirty people” or sinners, which supports perceived stigma among them, align with the previous reports on stigma and moral experiences [80,82], suggesting that the moral standing of an individual is influenced by the moral discourse of the community or a society and the requisite morally appropriate behaviour is the behaviour that can meet socially constructed discourse.

The study has several limitations that should be considered in the interpretation of its findings. Firstly, the study included a small number of respondents in a single setting, which could have led to a biased overview of HIV stigma and discrimination against married men living with HIV. The snowball technique used to recruit the participants may also be a limitation as it might have led to under-sampling of married men with HIV outside of the social networks of the current participants. Therefore, the findings of this study may be less likely to be transferable to other married men with HIV with different characteristics and in different settings in the country. However, these findings are useful to informing governments and organisations or institutions concerned with HIV, health service providers and program planners to develop evidence-based interventions that address the stigma and discrimination facing PLHIV within families, communities, and healthcare facilities. Future studies that cover heterogeneous participants with different backgrounds and from different settings, and that further explore religious thoughts and moral stances of participants in relation HIV infection, are recommended.

## 5. Conclusions

This study found that HIV-positive men married to women experienced external, anticipated and perceived HIV stigma and discrimination due to their HIV status. External stigma was experienced within healthcare facilities, communities and families where they lived and interacted, which was reflected in various discriminatory behaviours and treatments by healthcare professionals, community and family members. Anticipated stigma was based on the HIV-related stigma and discrimination experienced by other PLHIV, such as neglect, rejection, avoidance and burning of personal belongings, and on the perception that being infected with HIV is a disgrace, which can ruin the reputation of the extended family. Participants’ individual moral judgement that associated their HIV status with amoral behaviours and engagement in sex with female sex workers, and participants’ self-judgement as ‘dirty people’ or sinners which increased guilty feelings, were additional factors leading to HIV’s perceived stigma. The participants’ moral judgement also led them to endorse negative stereotypes regarding themselves or self-stigma. The current findings indicate the need for HIV stigma-related training for healthcare professionals, which has been reported to be effective for stigma reduction [35,83,84], and broader coverage of HIV information dissemination for people within families and communities to improve their knowledge on HIV, and mitigate stigmatising and discriminatory attitudes, behaviours or treatments and services for PLHIV within families, communities and healthcare facilities.

## Figures and Tables

**Table 1:** Socio-demographic characteristic of the participants.

Characteristics	No. of Respondents n = 20 (%)
Age	
<30	3 (15)
30–39	6 (30)
40–49	11 (55)
50–59	1 (5)
HIV diagnosis	
1 to 5 years ago	5 (25)
6 to 10 years ago	11 (55)
11 to 15 years ago	4 (20)
Province of origin	
Special Region of Yogyakarta	13 (65)
Central Java	2 (10)
Jakarta	4 (20)
Bengkulu	1 (5)
Education	
University graduates	5 (25)
Senior High school graduates	8 (40)
Junior High school graduates	4 (20)
Elementary school graduates	3 (15)
Occupation	
Supervisor in clothes shop	1 (5)
NGO workers (volunteers)	1 (5)
Café/restaurant waiter	2 (10)
Freelance photographer	1 (5)
Construction worker	1 (5)
Online motor-taxi drivers	3 (15)
Assistant in a motor-workshop (bengkel)	1 (5)
Entrepreneurs	7 (35)
Pet (birds) seller	1 (5)
Employees at private company	2 (10)
